# Limited genetic variations of the Rh5-CyRPA-Ripr invasion complex in *Plasmodium falciparum* parasite population in selected malaria-endemic regions, Kenya

**DOI:** 10.3389/fitd.2023.1102265

**Published:** 2023-03-01

**Authors:** Harrison Waweru, Bernard N. Kanoi, Josiah O. Kuja, Mary Maranga, James Kongere, Michael Maina, Johnson Kinyua, Jesse Gitaka

**Affiliations:** 1 Centre for Research in Infectious Diseases, Directorate of Research and Innovation, Mount Kenya University, Thika, Kenya; 2 Department of Biochemistry, Jomo Kenyatta University of Agriculture and Technology, Nairobi, Kenya; 3 Centre for Research in Tropical Medicine and Community Development, Nairobi, Kenya; 4 Department of Biology, University of Copenhagen, Copenhagen, Denmark; 5 Malopolska Centre of Biotechnology, Jagiellonian University, Kraków, Poland

**Keywords:** genetic variations, Rh5-CyRPA-Ripr invasion complex, malaria, vaccines, erythrocyte (RBC)

## Abstract

The invasion of human erythrocytes by *Plasmodium falciparum* merozoites requires interaction between parasite ligands and host receptors. Interaction of *Pf*Rh5-CyRPA-Ripr protein complex with basigin, an erythrocyte surface receptor, *via PfRh5* is essential for erythrocyte invasion. Antibodies raised against each antigen component of the complex have demonstrated erythrocyte invasion inhibition, making these proteins potential blood-stage vaccine candidates. Genetic polymorphisms present a significant challenge in developing efficacious vaccines, leading to variant-specific immune responses. This study investigated the genetic variations of the *Pf*Rh5 complex proteins in *P. falciparum* isolates from Lake Victoria islands, Western Kenya. Here, twenty-nine microscopically confirmed *P. falciparum* field samples collected from islands in Lake Victoria between July 2014 and July 2016 were genotyped by whole genome sequencing, and results compared to sequences mined from the GenBank database, from a study conducted in Kilifi, as well as other sequences from the MalariaGEN repository. We analyzed the frequency of polymorphisms in the *Pf*Rh5 protein complex proteins, *Pf*Rh5, *Pf*CyRPA, *Pf*Ripr, and *Pf*P113, and their location mapped on the 3D protein complex structure. We identified a total of 58 variants in the *Pf*Rh5 protein complex. *Pf*Rh5 protein was the most polymorphic with 30 SNPs, while *Pf*CyRPA was relatively conserved with 3 SNPs. The minor allele frequency of the SNPs ranged between 1.9% and 21.2%. Ten high-frequency alleles (>5%) were observed in *Pf*Rh5 at codons 147, 148, 277, 410, and 429 and in *Pf*Ripr at codons 190, 255, 259, and 1003. A SNP was located in protein-protein interaction region C203Y and F292V of *Pf*Rh5 and *Pf*CyRPA, respectively. Put together, this study revealed low polymorphisms in the *Pf*Rh5 invasion complex in the Lake Victoria parasite population. However, the two mutations identified on the protein interaction regions prompts for investigation on their impacts on parasite invasion process to support the consideration of *Pf*Rh5 components as potential malaria vaccine candidates.

## Background

The World Health Organization (WHO) estimates the latest global malaria health burden statistic at 627 000 deaths resulting from 241 million malaria infection cases (1). The scourge’s heaviest health and economic burden is borne by the developing countries in Sub-Saharan Africa, where an estimated 90% of all malaria deaths occur, with children under five accounting for 78% of all deaths (2). The emergence of multi-drug resistance *Plasmodium falciparum (P. falciparum)* resistant strains and insecticide resistance mosquitos remains a significant challenge in treating and eliminating malaria (3, 4). The lack of an effective vaccine remains one of the most critical gaps in the strategies developed to eliminate *P. falciparum* malaria (5).

The development of an effective *P. falciparum* vaccine focuses on targeting pre-erythrocytic or erythrocytic stages for parasite development and malaria pathogenesis in humans or the parasite development within the mosquito vector (6). Symptoms elicited by parasite infection originate from the erythrocytic stage of malaria infection. At this stage, the merozoite invades the erythrocytes, where at initial recognition of the human erythrocytes, the merozoite orients itself such that the apical region comes to direct contact with the host’s erythrocyte membrane, followed by irreversible attachment of merozoites to erythrocytes (7). Ring-like moving junction mediates complete parasite internalization to formation of an intracellular parasitophorous vacuole (8). The whole erythrocyte invasion process is mediated by multiple merozoite proteins mainly expressed on the surface, or in the apical organelles such as rhoptry and microneme (9). Since these proteins are essential for invasion and are exposed to host immune system, they are considered ideal targets for blood stage vaccines (BSV) (10–12). However, the exposure of candidate BSV antigens to human immune system during natural infections subjects them to selective pressure, that may result to high levels of polymorphisms (13). This presents a significant challenge for allele-specific immune responses as an ideal vaccine must be able to protect against multiple genetic variants of parasites (14, 15).

The *P. falciparum* reticulocyte binding homolog 5 complex (*Pf*Rh5) is a primary vaccine target for developing an effective malaria vaccine. The *Pf*Rh5 complex comprises four interacting proteins: *Pf*- reticulocyte binding homolog 5 (Rh5), *Pf*- interacting protein (*Pf*Ripr), *Pf*-Cysteine-rich protective antigen (CyRPA), and *Pf*-P113 protein (16). *Pf*Rh5 proteins bind to erythrocytes *via* the host receptor basigin, while the other three proteins interact within the complex to initiate erythrocyte invasion. *Pf*CyRPA binds directly to *Pf*Rh5, while *Pf*Ripr interacts with *Pf*CyRPA; thus, *Pf*CyRPA forms the contact sites for *Pf*Rh5 and *Pf*Ripr. Studies have shown that *Pf*P113 interacts with *Pf*Rh5 protein on the N-terminal, providing a releasable mechanism for anchoring *Pf*Rh5 to basigin (11, 17). The genes encoding for these proteins are highly maintained, as shown in gene knockout experiments suggesting they are vital for parasite survival (18, 19). Antibodies against *Pf*Rh5, *Pf*Ripr, and *Pf*CyRPA have been shown to inhibit parasite erythrocyte invasion in non-human primates and mice, while antibodies against *Pf*P113 protein have been associated with protection against clinical malaria *in vivo* (20, 21). These studies suggest that all proteins of the *Pf*Rh5 complex can be considered potential BSV targets.

Polymorphisms in all *Pf*Rh5 complex encoding genes could impede the development of an Rh5 malaria BSV. Like the *Pf*Rh5 complex, apical membrane antigen 1 (AMA1), once considered a potential malaria vaccine candidate is also essential for invasion. However, AMA1 is highly polymorphic, leading to allele-specific immune responses and limited efficacy in its Phase IIb trials (22). Investigation into polymorphisms on all members of the *Pf*Rh5 complex, their effects on the protein structure, and their association are significant considerations when designing a vaccine. Studies have demonstrated that *P. falciparum* parasites have a high within host genetic diversity in high transmission regions compared to low transmission settings (23, 24). This is due to the increased probability of recombination between genetically distinct variants in high transmission settings. This extensive genetic diversity is a major hindrance in malaria vaccine development as the host immune responses may fail to recognize all the variants of an antigen (25).

Here, to explore these questions, we analyzed the four genes of the *Pf*Rh5 complex by whole genome sequencing in a cross-sectional sample of parasites from two high malaria transmission regions in Kenya.

## Methods

### Sampling, DNA preparation, and whole genome sequencing

Parasite DNA was extracted from archived whole blood samples from patients recruited for a drug resistance surveillance study in local hospitals on four selected islands (Mfangano, Takawiri, Kibuogi, and Ngodhe) in Lake Victoria, a coastal mainland (Ungoye) between July 2014 and July 2016. The study’s approval was obtained from the Kenyatta National Hospital - University of Nairobi (KNH-UoN) ethical review committee (P609/10/2014) and the Mount Kenya University Ethics Review Committee (038/2014). Written consent was obtained from all the participants or guardians, and malaria cases were treated per the national malaria guidelines. The samples re-analyzed here were a subset of these studies which has been extensively described elsewhere (26, 27). Briefly, to increase the parasitemia, the field *P. falciparum* parasites were adapted for *in vitro* culture as previously described (28), and DNA was extracted from short-term cultures (1 month) at the schizont stage using QIAamp DNA mini kit (Qiagen, Valencia, CA). Paired-end sequencing libraries were prepared using Nextera XT DNA library preparation Kit according to the manufactures protocol. (Illumina, USA). Whole genome sequencing was performed on Illumina MiSeq technology (Illumina, USA) at 30X coverage generating reads of length 150 bps. These sequences are archived at the DDBJ BioProject, Accession number PRJDB12148. Quality control checks were performed using the FASTQC (Babraham Institute, UK) toolkit version 0.11.5.

### Comparison of polymorphisms identified with other regions

For comparative analysis, we obtained previously reported whole genome sequences *P. falciparum* isolates collected from Kilifi, a malaria endemic region in coastal Kenya (29, 30). The mined sequences were generated from two drug trial studies that were conducted between 2005 and 2008, and the sequences deposited in the GenBank repository under accession numbers *Pf*Ripr: MW597717-MW597776, *Pf*Rh5: MW597550-MW597609, *Pf*CyRPA : MW597610-MW597716, and *Pf*P113: MW597459-MW597549.

We also accessed the catalogue of genetic variation in *P. falciparum*, of the global MalariaGEN database v6.0, for comparing and validating the SNPs identified from the Lake Victoria sample population. This dataset comprised of genomic variation records of 7,113*P. falciparum* samples from 28 malaria-endemic countries. The method used to retrieve the data was previously described by Amato et al., 2016. The dplyr v1.0.9 package (Wickham H, François R, Henry L, 2022) in R v4.2.1 was used to filter out the four genes of the *Pf*Rh5 complex using their PlasmoDB unique identifiers. SNPs identified were then filtered and analyzed.

### Read mapping and coverage

Sequence reads were aligned against *Plasmodium falciparum* 3D7 reference genome (version 8.1) (https://plasmodb.org/common/downloads/release-46/Pfalciparum3D7/fasta/data/) using Burrows-Wheeler Alignment tool (BWA) (31) (http://bio-bwa.sourceforge.net) with default parameters. The resulting alignment was further processed with Samtools (32) and Picard v1.66 (33) to remove duplicates. SNPs were called using Genome Analysis Toolkit (GATK) HaplotypeCaller with the following parameters – genotyping mode DISCOVERY, –output mode EMIT_VARIANTS_ONLY, –stand_emit_conf 10, and – stand_call_conf 30. To improve the quality of variant calling, we further discarded genotyping calls with coverage of <5 reads. The resulting variant call format (VCF) files were then merged into one file using VCF tools (34).

### Variant calling and analysis

The VCF file containing twenty-six samples that passed the quality test from read mapping analysis was used as the input file in VCF tools for variant analysis. High-quality SNPs in four target genes, *Pf*Rh5, *Pf*CyRPA, *Pf*Ripr, and *Pf*P113, were functionally annotated in the SNPEFF tool (35). Called variants were further analyzed in ARTEMIS software (36). MEGA 7 tool was used to perform multiple sequence alignment and translation of nucleotide sequences to amino acid sequences. Gene variants were identified by aligning the amino acid sequence reads to their corresponding 3D7 reference gene sequence. To test sensitivity of the above approach, we analyzed the sequences at different variant calling parameters to assess the impact of these settings on downstream analysis. Additionally, prior to variant calling, we performed base quality score recalibration to adjust the base quality scores of sequencing reads as well as local realignment around indels to reduce false-positive variant calls resulting from alignment artifacts.

### Population genetics analysis

The population genetic tests for the neutral theory of evolution (37) and Tajima’s D and Fu & Li’s statistics and nucleotide diversity (Pi) were calculated using DnaSPv6.1 (38). Tajima’s D tested departure from neutrality based on allele frequency distribution in each gene. Fu and Li’s D test statistic calculated the variation between the observed number of singletons and the total number of mutations. Pi was used to test the genetic diversity of each gene of the *Pf*Rh5 complex within the parasite population from Lake Victoria region. The *P.falciparum* adenylosuccinate lyase gene, a house keeping gene and apical membrane antigen gene were used as control in this analysis. The sequences for these genes were obtained from Lake Victoria parasite population.

### Protein structures

The structure of the Rh5-CyRPA-Ripr complex was retrieved from the Protein Data Bank (http://www.rcsb.org/) under the protein ID 6MPV. The datasets generated from this study were used to map the polymorphic sites of *Pf*Rh5 and *Pf*CyRPA protein structures on the Rh5-CyRPA-Ripr complex 3D structure in Pymol (The PyMOL Molecular Graphics System, Version 2.2.0, Schrödinger, LLC) to determine the location of the SNPs in the 3D protein structure and whether the SNPs were localized in the protein-protein interaction regions of the complex.

## Results

### Genetic variation in the *Pf*Rh5 complex genes

**Figure 1:**
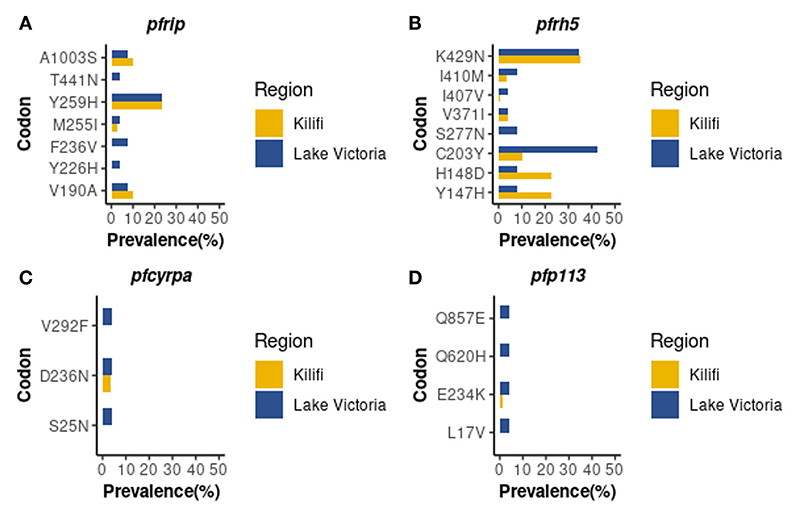
Prevalence of *PfRh5* complex polymorphisms. Frequency in percentage of single nucleotide polymorphisms identified across four genes of the Rh5 invasion complex **(A)** *Pf*Ripr, **(B)** *Pf*Rh5, **(C)** *Pf*CyRPA **(D)** *Pf*P113 in Lake Victoria (n=26) and Kilifi (n=68) populations.

Whole genome sequence analysis data for the four genes of the *Pf*Rh5 complex were obtained from 26 samples from the Lake Victoria islands.A total of 45, 35, 25, and 3 Non-synonymous SNPs were identified within the *Pf*P113, *Pf*Ripr, *Pf*Rh5, and in the *Pf*CyRPA genes, respectively. The minor allele frequency in the four genes ranged from 0.7% to 24.06%. High-frequency alleles (>5%) were identified in codons Y147, H148, C203, S277, I410, and K429 of the *Pf*Rh5 gene and codons V190, M255, Y259, and A1003 of the *Pf*Ripr gene (Figure 1). Non-synonymous SNPs identified in *Pf*CyRPA gene at codons S25, D236, and V292 and *Pf*P113 gene at codons L17, E234, Q620, and Q857 occurred at low frequency (Figure 1).

### Comparison of polymorphisms identified with the Kilifi population

A total of nine non-synonymous previously not observed from the Kilifi population were identified across the four genes in Lake Victoria isolates (39). *Pf*Ripr gene at codon Y226, F236, and T441, *Pf*Rh5 at codon S277, *Pf*P113 at codon L17, Q620, and Q857, and *Pf*CyRPA gene at codon S25 and V292 (Figure 1).

### Comparison of polymorphisms identified with global MalariaGEN

We further explored the MalariaGEN data to establish whether the variants identified were also present in the global database. We also screened for variants missed due to differences in analysis methodologies. This analysis confirmed that most of the variants observed from the Lake Victoria population had been previously observed elsewhere and deposited in the global MalariaGEN database giving confidence in our analysis methodology on the probability of missing variants (Table 1).

### Population genetics statistics

**Figure 2:**
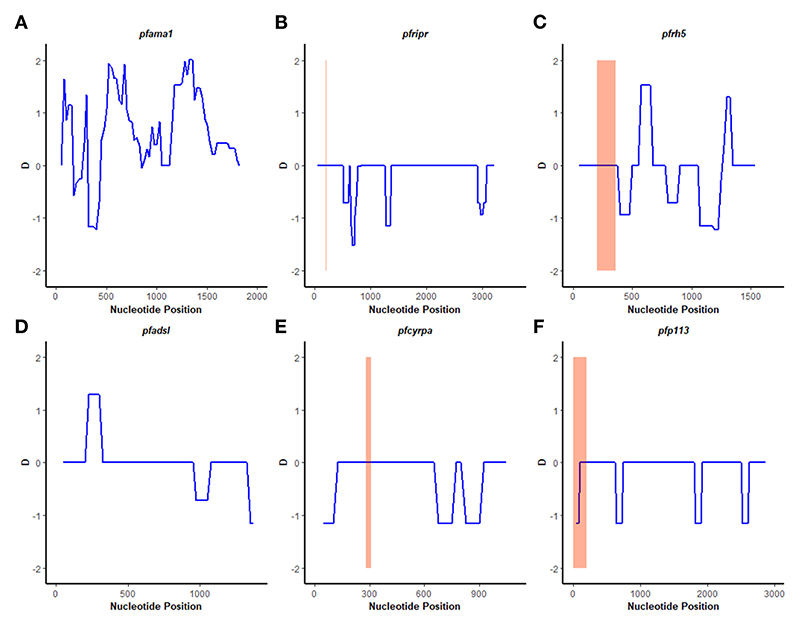
Tajima’s D analysis. Sliding window analysis of Tajima’s D test for neutrality for the four genes of the Rh5 invasion complex of twenty-six samples obtained from Lake Victoria region. **(A)** *Pfama1*, **(B)** *Pf*Ripr, **(C)** *pfrh5*, and **(D)** *pfadsl* **(E)** *pfcyrpa* **(F)** *pfp113* Tajimas’ D was calculated in DnaSP v6.1 software with a window length of 100 and a step size of 25 bases. D values are plotted against the mid-point of window length. The highlighted region indicates the basigin – *Pfrh5* binding site and protein-protein interactions regions for *Pfripr, Pfcyrpa*, and *Pfp113*.

**Figure 3:**
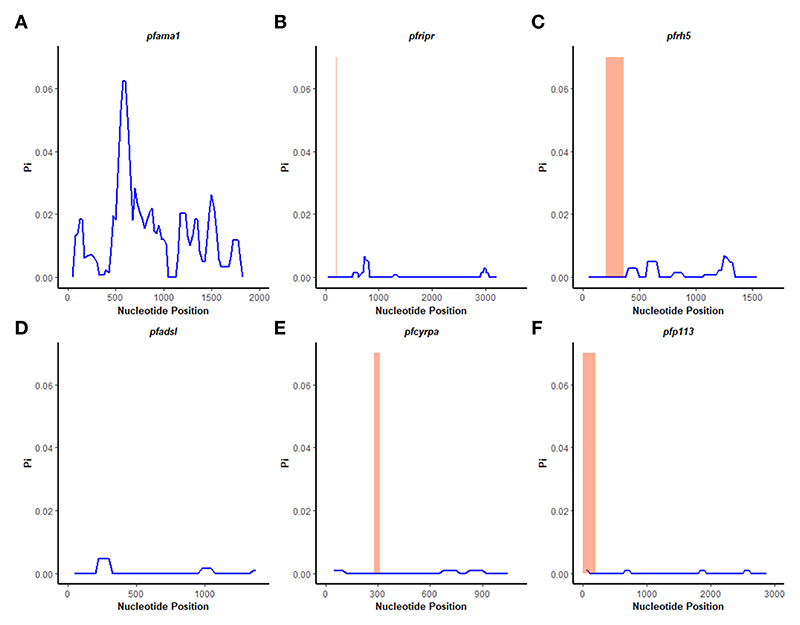
Nucleotide diversity analysis. Sliding window analysis of nucleotide diversity (Pi) per site to compare genetic diversity in four genes of the Rh5 invasion complex of twenty-six samples obtained from Lake Victoria region. **(A)** *pfama1*, **(B)** *pfripr*, **(C)** *pfrh5*, and **(D)** *pfadsl* **(E)** *pfcyrpa* **(F)** *pfp113*. Pi is nucleotide diversity calculated using DnaSP ver. 6.1 with a window length of 100 bases and a step size of 25 bases, plotted against the window length’s midpoint. The highlighted region indicates the basigin – PfRh5 binding residues and protein-protein interactions regions for PfRipr, PfCyrpa, and PfP113.

A sliding window approach was used to calculate the nucleotide diversity (*Pi*) and Tajima’s D statistics. All four genes had a negative neutrality summary statistic. Analysis revealed that the *Pf*P113 gene was the most conserved relative to the other three genes of the *Pf*Rh5 complex and the positive control *Pf*ama1 gene, with a Tajima’s D summary statistic of – 1.89 and a Pi of 0.00010 with 4 singleton mutations distributed along the 1578 bp nucleotide sequence. Relative to the negative control *Pf*adsl gene, the *Pf*Rh5 gene was the least conserved, with 25 non-synonymous polymorphisms distributed along the entire 2907 bp nucleotide sequence with a Tajima’s D summary value of -0.56 and a Pi of 0.00109 (Figures 2, 3). The Fu & Li’s statistics were not significant for *Pf*Rh5 and *Pf*Ripr genes. *Pf*CyRPA and *Pf*P113 gene had significant Fu & Li’s values, p < 0.05. (Table 2)

### Polymorphisms on the *Pf*Rh5 protein complex

**Figure 4:**
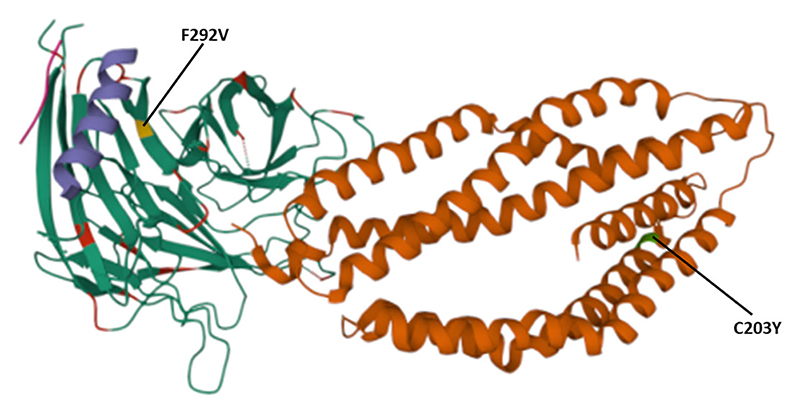
Location of polymorphisms on the 3D structure. Shows the SNPs identified that fall within the protein-protein and protein- basigin interaction regions of the Rh5 (brown), CyRPA (green) and Ripr (purple) protein complex. Polymorphic residues C203Y identified on Rh5 protein and F292V on CyRPA are highlighted in green and yellow, respectively. The Rh5 C203 mutation falls within the Basigin-Rh5 protein interaction region, while the CyRPA F292 mutation is located on blade 6, Ripr – CyRPA proteins interaction region.

The polymorphisms established from our dataset were mapped on the *Pf*Rh5 protein complex to show whether they occurred within known protein-protein interacting regions and the *Pf*Rh5 – basigin interaction region. The previously published Rh5-CyRPA-Ripr invasion complex (16) was superimposed with the basigin structure to show the interaction of *Pf*Rh5 with basigin. *Pf*Rh5 binds to basigin *via* His- 102 linker, α -2, α -4, and a disulfide loop (Cys345–Cys351) (40). The F350 and W447 *Pf*Rh5 residues stabilize binding by packing into basigin hydrophobic bonds. Only one SNP corresponding to codon 203 within the *Pf*Rh5-basigin interacting region was identified. *Pf*Ripr binds to *Pf*CyRPA blade 6 at amino acid residues 281 – 311. One mutation corresponding to this interaction region at codon 292 of *Pf*CyRPA was identified. No mutation corresponding to *Pf*CyRPA and *Pf*Rh5 binding regions was identified. Other polymorphisms were localized outside the protein interaction site (Figure 4).

## Discussion

*P. falciparum* infects and replicates in human host erythrocytes leading to manifestation of clinical of malaria. The invasion process by invasive merozoites involves the interaction of *Pf*Rh5 protein and the basigin receptor localized on the erythrocyte membrane (41, 42). However, *Pf*Rh5 does not function alone. Upon secretion, it forms a heteromeric complex with two micronemal proteins, *Pf*Ripr and *Pf*CyRPA. *Pf*Ripr and *Pf*CyRPA proteins do not interact with basigin and have been shown to lack a membrane anchor (11). The rationale for developing a blood-stage malaria vaccine targeting the components of the *Pf*Rh5 complex has been supported by *in-vitro* and *in-vivo* studies in non-human primates. Antibodies raised against the *Pf*Rh5 proteins have been shown to block erythrocyte invasion by inhibiting its binding to basigin receptor (5, 40, 43–45). Genes coding for proteins of the *Pf*Rh5 complex are highly conserved in *P. falciparum*, suggesting their vital role in parasite survival (30, 46). Therefore, an *Pf*Rh5-complex-based vaccine would prove effective. In the present study, we identified the genetic variations of the proteins that make up the *P. falciparum* Rh 5 complex and determined the polymorphism’s locus on the protein complex in the parasite population from the Mfangano, Takawiri, Kibuogi, and Ngodhe Islands of Lake Victoria in Western Kenya and compared with Kilifi and global databases. All genes of the *Pf*Rh5 complex were relatively conserved, and the negative population genetics statistic suggests the parasite population has limited potential to retain these mutations.

The observed negative Tajima’s D statistics from the Lake Victoria population indicated an excess of rare variants and do not suggest balancing selection (30). Genes with a significant negative Tajima’s D value indicate that the parasites population has a limited potential to retain polymorphisms, especially *Pf*P113 and *Pf*Ripr genes (47). These findings are consistent with previous studies of *P. falciparum* in the African population, which showed a majority of genes having a negative Tajima’s D value, suggesting a historical parasite population expansion event (48, 49).

In contrast to other merozoite antigens that are considered potential vaccine candidates such as Apical membrane antigen 1 (AMA1), merozoite surface protein 1 (MSP1), and merozoite surface protein 10 (MSP10) (50, 51), majority of polymorphisms of the *Pf*Rh5 complex components were rare variants and did not indicate balancing selection. Recent studies from Nigeria and Kenya reported one non-synonymous mutation on *Pf*Rh5 protein at codon C203Y (30, 52). We identified the C203Y mutation in the Lake Victoria population while mutations at codon Y147H, H148D, and K429N were reported in Kilifi samples and MalariaGEN global database as rare variants, which suggests a need for *P. falciparum* to maintain these mutations across various populations. Mutation at codon S277N observed from Lake Victoria isolates was not reported from the Kilifi population. Three singleton mutations at codons Y226H, F236V, and T441N of the *Pf*Ripr gene were identified in Lake Victoria. Among the three polymorphisms, only the mutation at codon Y226H was reported in MalariaGEN global dataset. The mutations were, however, absent from the Kilifi populations.

*P. falciparum* population from Uganda identified 16 SNPs in the *Pf*Ripr gene (53). Among the SNPs on P*f*Ripr gene identified in our study, three were also observed in Uganda, where a negative population statistic on these variants was reported (53). Considering the geographical proximity between Uganda and Lake Victoria islands, the common variants across the two study sites should be investigated to determine if they affect the functionality of the *Pf*Rh5 complex. Mutations on *Pf*CyRPA S25N, V292F, and *Pf*P113 gene L17V, Q620H and Q857E were identified only in Lake Victoria isolates.

We identified two mutations at the basigin-*Pf*Rh5 interaction region and *Pf*CyRPA-*Pf*Ripr proteins interaction regions. The mutation C203Y on *Pf*Rh5 protein was located on the Rh5- basigin interface, while mutation V292F located on blade 6 of *Pf*CyRPA protein which is the region of interaction with *Pf*Ripr. Studies have demonstrated that recombinant *Pf*Rh5 with the C203Y mutation binds to basigin with the same affinity as the wild type (54).

The components of the *Pf*Rh5 complex are located in different subcellular locations; thus, the complex only forms during erythrocyte invasion when they are secreted from the rhoptries or micronemes (42). Field studies have demonstrated the *Pf*Rh5 complex components exhibit low immunogenicity suggesting the antigens are under limited immune pressure (55). This could explain the limited high-frequency and rare variants observed in this study, as the parasite has a limited need to acquire mutations to escape host immune responses.

Put together, developing an effective malaria vaccine remains a priority among strategies to eliminate and eradicate the disease. One major hindrance to achieving this is the emergence of polymorphisms within the various vaccine antigen targets leading to allele-specific immune responses. Among the *Pf*Rh5 complex, *Pf*Rh5 is the most advanced vaccine target. However, the presence of low-frequency mutations raises concerns about immune system evasion. This study recommends functional assay studies to investigate the immunological and biological relevance of the identified mutations.

## Figures and Tables

**Table 1:** List of high frequency SNPs identified in Lake Victoria and Kilifi parasite population.

Gene	Position	Ref	Alt	S/NS	Lake Victoria (n=26)	Kilifi (n=68)	MalariaGEN global dataset (n=7,113)
***pf*ripr**	*pfripr_190*	V	A	NS	7.7	10.3	+
	pfripr_226*	Y	H	NS	3.8	0	+
	pfripr_236*	F	V	NS	3.8	0	-
	*pfripr_255*	M	I	NS	7.7	2.8	-
	*pfripr_259*	Y	H	NS	23.1	23.6	+
	pfripr_441*	T	N	NS	3.8	0	-
	*pfripr_1003*	A	S	NS	7.7	10.3	+
***pf*rh5**	*pfrh_147*	Y	H	NS	7.7	22.6	+
	*pfrh_148*	H	D	NS	7.7	22.6	+
	*pfrh_203*	C	Y	NS	42.3	10.3	+
	*pfrh_277*	S	N	NS	7.7	0	-
	pfrh_371*	V	I	NS	3.8	4.1	+
	pfrh_407*	I	V	NS	3.8	0.7	+
	*pfrh_410*	I	M	NS	7.7	3.4	+
	*pfrh_429*	K	N	NS	34.6	34.9	+
***pf*cyrpa**	pfcyrpa_25*	S	N	NS	3.8	0	-
	pfcyrpa_236*	D	N	NS	3.8	3.4	-
	pfcyrpa_292*	V	F	NS	3.8	0	+
***pf*p113**	pfp113_17*	L	V	NS	3.8	0	-
	pfp113_234*	E	K	NS	3.8	1.4	+
	pfp113_620*	Q	H	S	3.8	0	-
	pfp113_857*	Q	E	NS	3.8	0	-

Polymorphisms identified in both populations and the global MalariaGEN dataset are indicated with a plus +, and singleton SNPs are indicated by an asterisk*.

Ref refers to the 3D7 *Plasmodium falciparum* strain reference amino acid, while Alt refers to the amino acid variation. The SNPs are classified under S (synonymous or NS (non-synonymous) and frequency of SNPs is expressed as percentages.

**Table 2:** Fu & Li’s neutrality tests statistics based on Lake Victoria sample population.

Gene	No. of Samples	S	Fu & Li’s D*	Fu & Li’s F*
***pfcyrpa***	26	3	-2.58495*	-2.7088*
***pfp113***	26	4	-2.86849*	-2.99593*
***pfrh5***	26	8	0.10999	-0.10381
***pfrirp***	26	7	-1.11002	-1.37673
***pf*** **ama1**	26	38	4.28469	3.98709
***pf*** **adsl**	26	3	-0.21602	-0.25135

S refers to segregation sites. Asterisk * indicates significance, p > 0.05.

## Data Availability

The datasets presented in this study can be found in online repositories. The names of the repository/repositories and accession number(s) can be found below: https://www.ddbj.nig.ac.jp/, PRJDB12148.
